# Evaluating the efficacy of hinged elbow braces in reducing passive valgus forces after ulnar collateral ligament injury—A biomechanical study

**DOI:** 10.1002/jeo2.70094

**Published:** 2025-01-03

**Authors:** Kai Hoffeld, Christopher Wahlers, Jan P. Hockmann, Sebastian Wegmann, Nadine Ott, Kilian Wegmann, Lars Peter Müller, Michael Hackl

**Affiliations:** ^1^ Department of Orthopaedic, Trauma and Plastic Surgery, Faculty of Medicine and University Hospital of Cologne University of Cologne Cologne Germany; ^2^ Orthopädische Chirurgie München Munic Germany; ^3^ Department of Orthopaedic and Trauma Surgery, University Medical Centre Mannheim, Medical Faculty Mannheim University of Heidelberg Mannheim Germany

**Keywords:** elbow brace, elbow orthosis, UCL injury, UCL rupture, valgus instability

## Abstract

**Purpose:**

This biomechanical study aimed to investigate the effectiveness of a hinged elbow orthosis in reducing passive valgus forces following medial ulnar collateral ligament (UCL) injuries of the elbow joint. The hypothesis tested was that a hinged elbow orthosis reduces these passive valgus forces.

**Methods:**

Eight fresh frozen cadaveric elbow specimens were prepared and tested under three scenarios: intact ligaments, simulated UCL rupture and application of a hinged elbow brace after simulated UCL rupture. Valgus instability was assessed using a custom testing set‐up and the Optotrak motion capture system. Statistical analysis was conducted to compare the results across scenarios.

**Results:**

Intraclass correlation (ICC) calculation showed that the testing set‐up was reliable in investigating valgus deflection across all levels of applied force. The hinged elbow brace reduced passive valgus forces after UCL rupture. The reduction in valgus instability was consistent with close approximation to the native state, although not reaching its level.

**Conclusion:**

The hypothesis—that a hinged elbow orthosis significantly reduces passive valgus forces in the elbow following UCL injuries—is not supported by the data and therefore has to be rejected. Nevertheless, the study demonstrates a tendency that a hinged elbow brace could mitigate these forces, at least in an experimental cadaveric model with static study conditions.

**Level of Evidence:**

The level of evidence of this study is level IV.

**Abbreviations**
CI: confidence interval
ICC: intraclass correlation
K‐wire: Kirschner wire
MCL: medial collateral ligament
UCL: ulnar collateral ligament

## INTRODUCTION

Acute injuries of the medial ulnar collateral ligament (UCL) of the elbow are commonly reported in throwing or overhead athletes like baseball, tennis, volleyball or javelin throwers [6], but are also seen in simple elbow dislocations [22] and fracture dislocations [21].

Tears of the UCL are commonly treated conservatively with braces, tapes and physiotherapy, with avoidance of valgus stress [20, 21]. Surgical treatment is an option for patients with gross instability and/or chronic instability with functional impairment after conservative treatment [6, 21]. Various rehabilitation protocols have been developed to ensure joint congruity and at the same time restore mobility as quickly as possible [21, 26]. To protect against valgus stress, both conservative and post‐operative treatment protocols include the use of a hinged elbow brace [20, 21, 26]. In contrast to the abundant evidence available on the basics of the injury and its various treatment options, there is only very little evidence regarding the use of hinged braces. Biomechanical studies conducted by Manocha et al. suggested that hinged braces are unlikely to provide additional stability during dynamic range of motion in various positions of the elbow, neither after lateral collateral ligament [12] nor UCL injuries [13]. On the other hand, there is one biomechanical study that reported improved valgus stability after UCL injury when a hinged elbow orthosis was applied [19]. Besides the few mentioned studies, there is no further evidence about the absence or presence of a possible protective effect of hinged braces against valgus stress after UCL tears of the elbow. In addition, the protection against passive valgus forces in particular has not yet been sufficiently investigated, which represents an essential factor for successful rehabilitation after UCL injuries. Therefore, the aim of the present study was to investigate the effectiveness of a commercially available elbow orthosis in reducing passive valgus forces after UCL injury and thus to substantiate or invalidate its relevance in the treatment of this pathology. Consequently, the hypothesis of the study is that a hinged elbow orthosis in a 90° fixed flexion position significantly reduces passive valgus forces in the elbow following UCL injuries, while the null hypothesis posits that the orthosis does not have a significant effect on reducing these forces.

## METHODS

### Ethical considerations

This in‐vitro study was approved by the local institutional review board (Ethical Committee of the Medical Faculty of the University of Cologne—VT (No: 21‐1454)). This study followed the guidelines for experimental investigation with human subjects required by our institution.

### Specimen preparation

For this biomechanical study, eight fresh frozen cadaveric elbow specimens from three male and five female body donors were available. This sample size was based on previous studies with similar designs [12, 13]. Three of the specimens were right arms and five of them were left arms. The mean age at the time of death was 82 years (min. 65 years, max. 87 years). The specimens were stored at −20°C and thawed at room temperature 16–18 h before dissection and biomechanical testing. Fluoroscopic and clinical examinations were performed to exclude specimens with osteoarthritis or signs of previous surgery and trauma. The soft tissue of the proximal humerus and the forearm was preserved.

### Biomechanical testing set‐up

**Figure 1:**
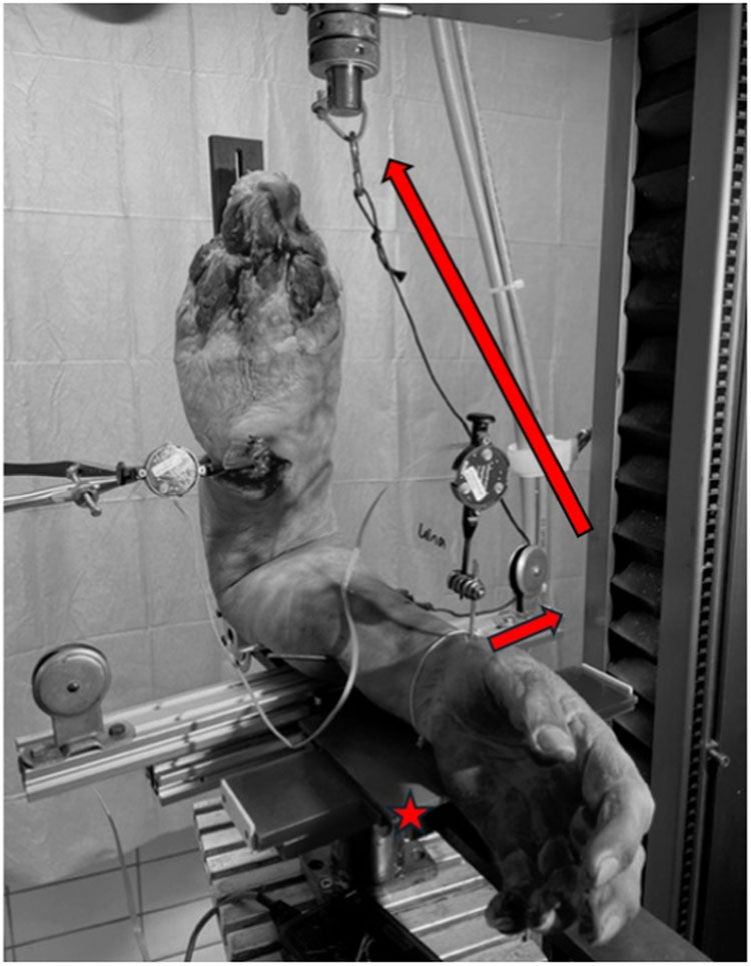
Upward movement of the mobile traverse (red arrows) resulted in valgus force and consecutive horizontal movement of the forearm on the sliding platform (red star).

**Figure 2:**
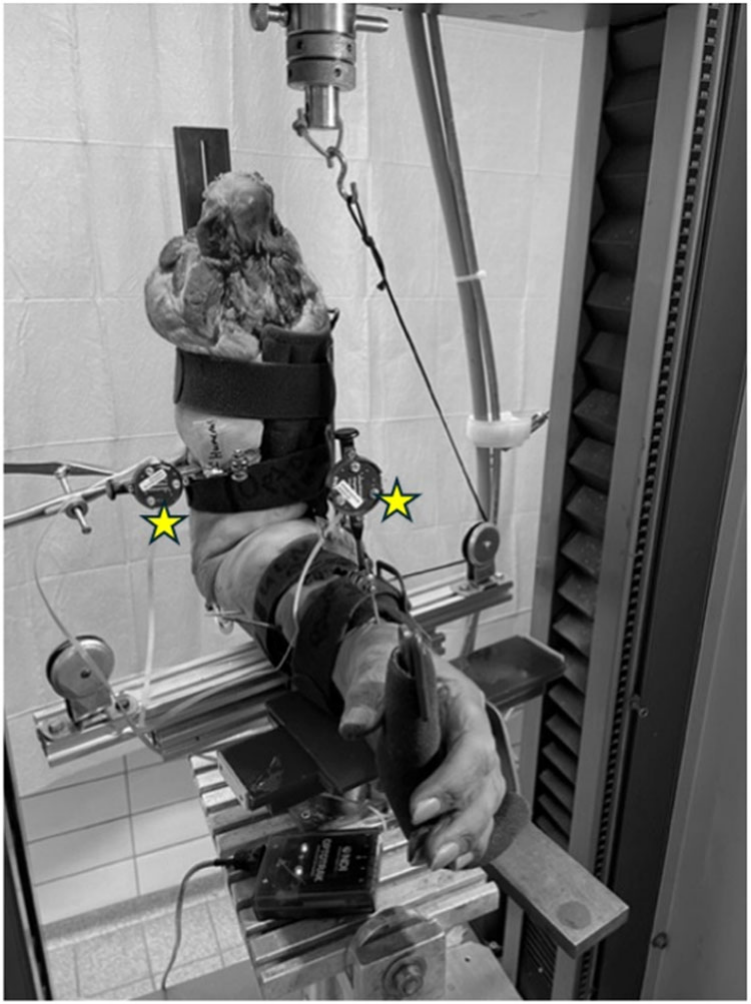
Arm with hinged elbow brace; position markers of the Optotrak® system are marked as yellow stars.

The humeral shaft was secured to a custom‐made testing fixture with an external fixator construction and one additional mounting clamp. The hinged testing fixture was positioned at a 90° angle and was mounted onto a servo‐hydraulic universal testing machine (ZwickRoell). The tested forearms of the specimens were secured in neutral position by two Kirschner wires (K‐wires), which were placed through the radius and the ulna. A mounting bolt was securely fixed to the lateral side of the ulnar shaft 10 cm distal to the centre of rotation. A synthetic wire connected the bolt to the mobile traverse of the testing machine. Reels were used for deflection of the wire. Thereby, upward movement of the mobile traverse resulted in a valgus force. By placing the forearm on a mobile platform, valgus forces could be converted into corresponding horizontal movements (Figure 1). A variant of this biomechanical testing set‐up was used in previous studies [9, 10, 16, 17]. To detect the differences in valgus instability we tracked the horizontal movement of the forearm by using the Optotrak Certus® motion capture system (Northern Digital Inc.). It is a camera‐based tracker, which captures the coordinates of position markers consisting of infrared light‐emitting diodes. The three sensors in a camera are placed slightly apart, so an active marker will be seen by the sensors at slightly different angles. For each marker, these two‐dimensional (2D) sensor data are used to calculate the 3D position coordinates. For data acquisition from the Optotrak system, we used Northern Digital's own ‘First Principles’ software. The corresponding sensor was attached to the K‐wire, which was drilled through the ulnar and radial shaft approximately 20 cm distal to the centre of rotation (Figure 2). All examinations were carried out in 90° flexion.

The testing protocol provided for an initial load of 1 N tensile force from the hydraulic machine. Positioning the traction pin on the lateral ulna approximately 10 cm distal to the centre of rotation of the elbow resulted in a traction force of 0.1 N m on the UCL. As a result, the elbow was always brought into the same starting position. The tensile force was then gradually increased to 10, 20 and finally 30 N, resulting in a valgus force of 1, 2 and 3 N m. The horizontal movement of the forearm was detected with the Optotrak system at all four levels, that is, the starting position, at 1, 2 and 3 N m. To test the effectiveness of the hinged brace in reducing valgus forces, three different scenarios (A–C) were prepared. The described testing protocol was repeated three times for each scenario and for each torque three times.

### Scenario A

In Scenario A, the skin, subcutaneous tissue, ligaments and fascia of the forearm remained intact (Figures 1 and 2). The cadaveric elbows were mounted on the testing set‐up as described above, and valgus instability was tested.

### Scenario B

**Figure 3:**
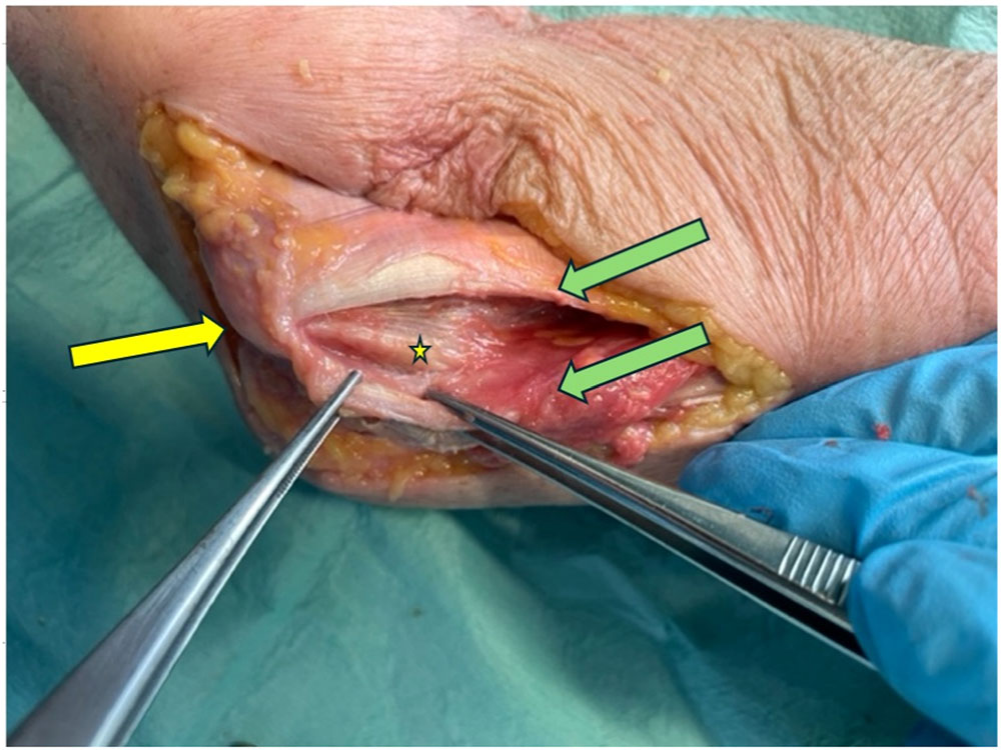
Specimen preparation of a right elbow: after performing the medial approach to the elbow with a split of the flexor muscles (green arrows) the UCL emerges (yellow star); medial epicondylus of the distal humerus marked with yellow arrow. UCL, ulnar collateral ligament.

After testing the specimens in the native state, a medial approach to the elbow was performed (Figure 3). The flexors were split but preserved, the posterior and anterior bundles of the UCL were detached from their humeral origin together with the medial capsule, mimicking a ruptured state of the UCL, resulting in valgus instability of the elbow. Valgus instability was confirmed through a clinical examination of the specimen elbow. This involved applying a valgus force to the elbow joint while observing the medial joint opening. The clinical examination was performed by a trained orthopaedic resident who assessed the medial joint line for increased laxity and abnormal movement, indicating instability. This was followed by testing for valgus instability as described above.

### Scenario C

After testing the specimens in the transected state, we applied a hinged elbow brace in a 90° fixed flexion position (medi Epico ROM®s, medi GmbH & Co. KG). The orthosis was secured to the arm using the adjustable strap system and a handpiece designed for a snug fit. This allowed easy adjustment and a custom fit for every specimen tested. The orthosis was positioned in such a way that the centre of rotation of the orthosis was projected onto the idealised centre of rotation of the elbow. According to Graham, the trochlea centre and the centerline of rotation are essentially in line with each other with the elbow flexed 90° [8]. Therefore, the centre of the trochlea was visualised in 90° flexion under fluoroscopy and the elbow orthosis was fitted in such a way that the hinge of the orthosis was in line with the trochlea. Then, the elbow was tested again for valgus instability as described below.

### Vector analysis

After gathering the three‐dimensional (3D) position coordinates from the Optotrak system for each step of the test protocol, we had each *X*, *Y* and *Z* position of the distal pin and thus we were able to calculate the horizontal movement, that is, the extend of the valgus instability, as mathematical vectors.

$$\vec{\upsilon }=\left(\begin{array}{l} x\\ y\\ x \end{array}\right).$$

$$|\vec{\upsilon }|=\sqrt{x^{2}+y^{2}+z^{2}}.$$

We tested every specimen for every scenario and every level of valgus force three times (i.e., three test repetitions) yielding three different vectors. The mean values of these three vectors were calculated and used in all statistical analyses. The calculated amounts of the vectors were given in millimetres. The vector length in millimetres refers to the horizontal movement of the position marker and represents the corresponding (smaller) movement that occurs due to the medial instability at the medial side of the joint.

The relationships between the scenarios and the corresponding summed vectors were then calculated as factors, that is, B/A and C/B as relative deviations, corresponding to a multiplicative model.

### Statistical analysis

The data collected were analysed using the Statistical Package for the Social Sciences statistical programme. Normal distribution was tested by Kolmogorov–Smirnov. We report the median (interquartile range). To determine differences between the groups we performed a two‐way repeated measures analysis of variance. The effect size was calculated according to Cohen. A *p* ≤ 0.05 was considered to be statistically significant. Intra‐specimen variability was measured using the intraclass correlation (ICC) according to Shrout and Fleiss [23]. ICC benchmarks were used as proposed by Cicchetti (poor: ICC < 0.4; moderate: 0.4–0.59; good: 0.6–0.74 and excellent: 0.75–1.0) [3].

## RESULTS

**Figure 4:**
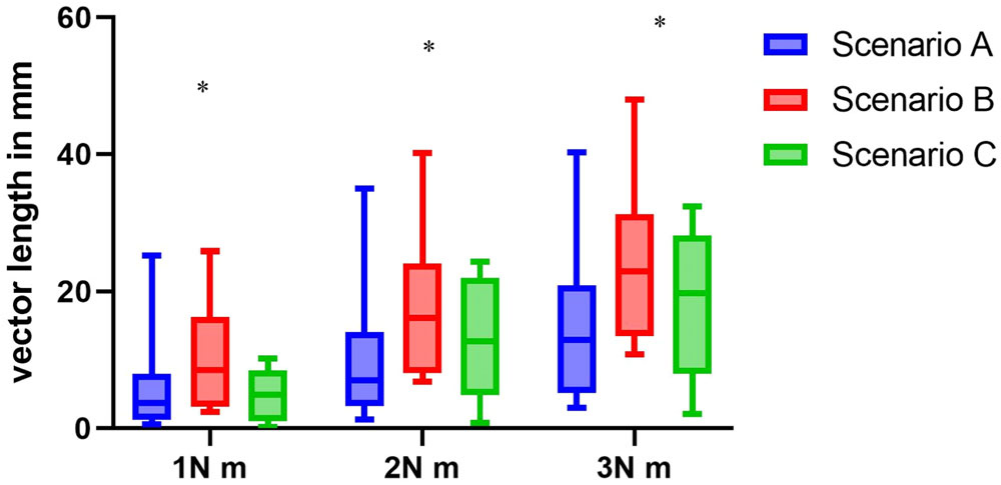
Valgus instability depending on the torque applied. Each box represents the interquartile range (from 25th to 75th percentiles), within which 50% of the values are contained. The line horizontally crossing each box represents the median. The error bars show the minimum and maximum values. A two‐way ANOVA for repeated measures was used to test for differences between the groups; significant differences are indicated with a ‘*’. ANOVA, analysis of variance.

The pooled mean value for the magnitude of the vector length is shown in Table 1. The smallest vector length was in Scenario A at 1 N m. The highest vector length was in Scenario B at 3 N m. There was a significant difference between the vector lengths within Scenarios B and C. The post hoc test showed that in Scenarios B and C, only the difference between 1 and 3 N m was significant (*p* = 0.041 and *p* = 0.014). For an overview of these results, see Table 1 and Figure 4.

**Table 1:** Mean vector lengths of the eight specimens in mm per scenario and applied force.

	Vector length in mm			
Applied force	Scenario A	Scenario B	Scenario C	*p*
1 N m	3.67 [1.22–7.99]	8.47 [3.17–16.26]	4.92 [1.03–8.46]	n.s.
2 N m	7.01 [3.24–14]	16.13 [8.03–24.14]	12.68 [4.83–22.01]	n.s.
3 N m	12.91 [5.14–20.93]	22.91 [13.44–31.16]	19.79 [7.99–28.1]	n.s.
*p*	n.s.	0.045	0.015	

*Note*: Data are reported as median [IQR]; a two‐way ANOVA for repeated measures was used to test differences between the scenarios and applied forces.

Abbreviations: ANOVA, analysis of variance; IQR, interquartile range.

The mean values of the vector lengths for every single specimen, scenario and torque are shown in Table S1.

The ratio of the vector length of Scenario A in comparison to Scenario B was on average 0.63 (95% CI: 0.47–0.78). The ratio between Scenarios B and C was on average 2.77 (95% CI: 1.42–4.13). Comparing Scenario A with Scenario C, the ratio was on average 2.07 (95% CI: 0.63–3.51).

The vector length ratios were statistically significantly different depending on the applied force (*p* = 0.038), but not for the respective scenario (n.s.). There was no significant influence of the interaction between the scenario and the force (n.s.).

The ICC for the intra‐specimen variability is depicted in Table 2.

**Table 2:** Intraclass correlation (ICC) for all specimens.

	ICC	95% CI	*p*
Specimen 1	0.895	0.663–0.974	<0.001
Specimen 2	0.995	0.983–0.999	<0.001
Specimen 3	0.806	0.410–0.951	<0.001
Specimen 4	0.795	0.358–0.949	<0.001
Specimen 5	0.914	0.337–0.983	<0.001
Specimen 6	0.986	0.9–0.997	<0.001
Specimen 7	0.957	0.843–0.99	<0.001
Specimen 8	0.747	0.273–0.936	0.002

Abbreviation: CI, confidence interval.

## DISCUSSION

The main finding of this study is that the hypothesis—that a hinged elbow orthosis significantly reduces passive valgus forces in the elbow following UCL injuries—is not supported by the data and, therefore, has to be rejected. Although there was a tendency for the brace to mitigate valgus forces, this effect did not reach statistical significance. The results show that the dissection of the UCL created an ulnar instability with increased horizontal deflection by 37%. The results further show a tendency, that when applying a hinged elbow brace to the elbow with valgus instability, the horizontal deflection was reduced. While the results presented in Table 2 suggest a large inter‐specimen variability, the calculation of the ICC for the intra‐specimen variability showed values of excellent quality in seven of the eight specimens. This shows the validity of the experiment carried out to record the effect of valgus instability and its change. Therefore, it is possible that the inter‐specimen variability is due to the different biological nature of the specimens available for testing.

The evidence about the effectiveness of elbow orthoses to reduce valgus instability remains controversial. One biomechanical study reported by Pincivero et al., involved designing a custom orthosis for a javelin thrower with unilateral MCL insufficiency. The authors applied varying degrees of valgus force to the athlete's elbow. They found that the hinged elbow orthosis restored valgus stability on both the injured and uninjured sides, although its effectiveness decreased as the valgus force increased [19]. In contrast, there is a biomechanical cadaver study in which the authors used a custom‐built simulator to study seven cadaver elbows in active and passive motion in the physiological state, with a torn UCL and with an elbow brace attached to the elbow with a torn UCL [13]. The authors found that the hinged elbow brace had no beneficial effects on valgus instability during passive movement and even increased valgus instability during active movement. In that experiment, the elbow fulfilled the whole range of motion from full extension to full flexion, active and passive. The authors addressed the issue that as the elbow flexes from a fully extended position, the humerus undergoes internal rotation. As the elbow approaches full flexion, the humerus externally rotates, causing the elbow to shift from a more valgus to a more varus position during flexion [5, 13]. Under these circumstances, the hinged elbow brace did not support valgus stability. Our data, on the other hand, demonstrate contradicting findings. The scenarios in our study were designed with a fixed 90° flexion position of the elbow. Valgus force of 1–3 N m was applied so that this reflects the approximate forces that can be assumed in light everyday movements, such as sliding an object or drinking [7]. The presented data show that in these specific situations, with rather a static movement without full flexion or full extension and with just a little force applied to the elbow, the hinged elbow brace may offer additive support of the medial elbow. Although the amount of force reduction does not reach the level of the native UCL, there is an indication of reduction with a close approximation to the native state. However, it is important to note that the brace was fitted perfectly every time under the study conditions. Therefore, the results can only show the protective effect under these circumstances. It is known from clinical practice that braces can slip out of place or be unintentionally put on incorrectly by patients. This may lead to a malalignment of the mechanical axis of the orthosis and the anatomic flexion–extension axis of the elbow, which may exert harmful leverage forces on the elbow joint [11]. This was also discussed as a possible reason for an increased valgus instability with an applied hinged elbow orthosis during active movement after UCL rupture in the previously mentioned cadaveric study by Manocha et al. [13]. The problem of harmonising the mechanical axis of the orthosis and the anatomical axis of the elbow could be further complicated by the fact that the flexion–extension axis of the elbow is altered by a UCL injury [1, 4]. However, the results of our study show that if the mechanical axis of the orthosis and the anatomical axis of the elbow are brought into alignment, the orthosis could have possibly a protective effect against valgus forces. The risks of malalignment might be avoided by using a handrest as well as an adequate introduction to brace application by the orthopaedic technician. It has also been shown that after surgical reconstruction using modern surgical techniques of the UCL, the anatomical flexion–extension axis is restored to approximately the physiological state and is no longer subject to variability as described above [2]. Therefore, complications due to an altered axis of the elbow following a UCL rupture should be mitigated after surgical treatment and the alignment of the mechanical axis of the orthosis with the elbow axis should be facilitated. This could indicate an increased efficacy or relevance of orthotic therapy after surgical treatment compared to the use of a hinged elbow brace in conservative therapy after a UCL injury. Beyond that, an improved orthosis design that ensures the correct alignment of the axes could improve the treatment with orthoses, which has also been suggested by other authors [13].

In addition to the lack of support of the orthoses for the elbow with valgus instability during movement of the joint, the previous biomechanical study by Manocha et al. showed a reduction of valgus instability due to active movement of the muscles [13]. This stresses the necessity of correct execution of exercises and movement patterns in general after UCL injuries. Systematic reviews and meta‐analyses in the field of knee surgery have shown that neuro‐muscular status plays a key role in the primary and secondary prevention of ligamentous knee injuries [14, 18]. Various training programmes to improve neuro‐muscular functionality have shown their effectiveness in preventing these injuries. There are no equivalent examinations for ligamentous injuries of the elbow. However, muscle training is a key element of rehabilitation programmes in elbow surgery [24, 26], because the flexor and extensor muscles of the forearm are secondary stabilisers of the elbow joint [15]. Hence, similar effects, like in the prevention of knee injuries, can be surmised. Therefore, we do not see our study results as contradicting the previous findings on the effects of a hinged elbow brace during elbow joint movement, but rather see our study as a completely different area of interest. While the effects mentioned above are important during rehabilitation programmes, protection from passive valgus forces is key for supporting healing during daily life. Most post‐operative protocols as well as the non‐operative protocols suggest non‐weight bearing or pausing the causative activities and different kinds of restriction of movement for at least six weeks [20, 24, 26]. While patients can protect their injured elbow in this period of time from valgus forces through conscious movement sequences, it can still happen repeatedly in everyday life that some movements of daily life are carried out less consciously or completely unconsciously. This may result in passive valgus forces affecting the elbow. The data presented in this study suggests that an elbow orthosis, at least in a 90° fixed flexion position, may reduce these described passive forces to the elbow in these situations of daily life, when the orthosis is in correct position and the mechanical and anatomical axis are correctly aligned.

## LIMITATIONS

The present study has several limitations. First, the small sample size may harbour the risk of a type two error. This is a well‐known and common drawback of biomechanical research. The sample size of eight cadaveric elbows was chosen to balance the feasibility of obtaining and preparing fresh frozen specimens with the need for sufficient data to test differences in valgus instability across the test scenarios. This number aligns with previous biomechanical studies that have demonstrated reliable and reproducible results using similar sample sizes [12, 13]. The chosen sample size also considers the high cost and limited availability of cadaveric specimens, making it a practical and justifiable choice for this study. Second, this study as well as most biomechanical studies face the problem of a big age difference of the older cadaver samples compared to the usually much younger patient population of interest. In addition, a disadvantage of this study is that the experiment was carried out in 90° flexion only. Other angular dimensions were not examined. The contradictory high vector length observed in the last specimen for scenario A could be due to pre‐test instability, tracker mobilisation, or specimen fixation failure, all of which could have led to erroneous measurements. Another limitation is the measurement error of the Optotrak system used. The accuracy provided is 0.1 mm although there could be a difference to the actual vectors and the vectors measured [25]. Finally, biomechanical study conditions do not accurately reflect the physiological reality that is to be investigated in the studies.

## CONCLUSION

The hypothesis—that a hinged elbow orthosis significantly reduces passive valgus forces in the elbow following UCL injuries—is not supported by the data and therefore has to be rejected. Nevertheless, the study demonstrates a tendency that a hinged elbow brace may reduce passive valgus forces following UCL rupture, thereby possibly offering a potential protective effect against valgus instability in specific static conditions. While there is variability among specimens, the consistent intra‐specimen results validate the experimental set‐up. The study highlights the importance of proper brace alignment with the elbow's anatomical axis to maximise efficacy. However, the protective effect is context‐dependent and may not extend to dynamic movements, emphasising the need for patient education and possibly improved orthosis design.

## AUTHOR CONTRIBUTIONS

**Kai Hoffeld**: Data curation; formal analysis; investigation; writing—original draft. **Christopher Wahlers**: Data curation; formal analysis; investigation; software. **Jan P. Hockmann**: Data curation; formal analysis; software; visualisation; writing—review and editing. **Sebastian Wegmann**: Project administration; resources. **Nadine Ott**: Funding acquisition; methodology; resources. **Kilian Wegmann**: Conceptualisation; funding acquisition; resources; writing—review and editing. **Lars Peter Müller**: Supervision; validation; writing—review and editing. **Michael Hackl**: Conceptualisation; methodology; supervision; writing—review and editing.

## CONFLICT OF INTEREST STATEMENT

The authors declare no conflicts of interest.

## ETHICS STATEMENT

Ethical approval for this study was given by the Institutional Review Board of the University Cologne (VT (No: 21‐1454)). Due to cadaveric character of this study, no informed consent was needed.

## Supporting information

Supplementary Information

## Data Availability

The data that support the findings of this study are available from the corresponding author upon reasonable request.
