# Supplementary material for: Evaluating the efficacy of hinged elbow braces in reducing passive valgus forces after ulnar collateral ligament injury—A biomechanical study
Source: J Exp Orthop. 2025 Jan 3;12(1):e70094. doi: 10.1002/jeo2.70094 (PMC11696245; doi:10.1002/jeo2.70094)
Supplement: Supplementary file 1 — Supplementary Information [file JEO2-12-e70094-s001.docx]

Table S1 Vector length in mm per applied force for every specimen

|  |  | **Vector length in mm** | | |
| --- | --- | --- | --- | --- |
| **Specimen** | **Applied force** | **Scenario A** | **Scenario B** | **Scenario C** |
| **1** | 1 N m | 4.1 | 3.1 | 1.6 |
|  | 2 N m | 7.0 | 6.8 | 8.5 |
|  | 3 N m | 9.1 | 10.8 | 12.5 |
| **2** | 1 N m | 2.6 | 2.4 | 0.2 |
|  | 2 N m | 7.0 | 8.1 | 0.7 |
|  | 3 N m | 16.5 | 17.1 | 2.1 |
| **3** | 1 N m | 0.6 | 7.5 | 2.4 |
|  | 2 N m | 1.2 | 13.9 | 9.2 |
|  | 3 N m | 3.0 | 18.8 | 15.7 |
| **4** | 1 N m | 3.2 | 18.4 | 10.2 |
|  | 2 N m | 6.4 | 25.5 | 21.0 |
|  | 3 N m | 9.3 | 31.5 | 25.0 |
| **5** | 1 N m | 7.9 | 9.5 | 8.5 |
|  | 2 N m | 15.0 | 18.4 | 16.2 |
|  | 3 N m | 21.7 | 27.0 | 23.9 |
| **6** | 1 N m | 0.7 | 3.3 | 0.8 |
|  | 2 N m | 2.2 | 8.0 | 3.6 |
|  | 3 N m | 3.8 | 12.2 | 6.5 |
| **7** | 1 N m | 8.0 | 9.7 | 7.4 |
|  | 2 N m | 11.0 | 20.1 | 22.3 |
|  | 3 N m | 18.5 | 30.1 | 32.4 |
| **8** | 1 N m | 25.2 | 25.8 | 8.5 |
|  | 2 N m | 34.9 | 40.2 | 24.4 |
|  | 3 N m | 40.3 | 48.0 | 29.1 |

All displayed vector lengths are mean values of three test repetitions
